# DAILY STRESSOR CONTROL AND AFFECTIVE REACTIONS: THE UNIQUE ROLE OF INTERPERSONAL STRESS

**DOI:** 10.1093/geroni/igad104.1327

**Published:** 2023-12-21

**Authors:** Dakota Witzel, Eric Cerino, Zachary Taylor, David Almeida

**Affiliations:** Penn State University, State College, Pennsylvania, United States; Northern Arizona University, Flagstaff, Arizona, United States; Northern Arizona University, Flagstaff, Arizona, United States; The Pennsylvania State University, State College, Pennsylvania, United States

## Abstract

Perceived control over daily stressors varies across life and stressor domains, but little is known about the function of perceived control as a protective resource for the impact of daily stressors on affective well-being. Using the third wave of the National Study of Daily Experiences (NSDEIII; N=1,263, Mage=62.62, 57.20% women), we examined how stressor control across domains (interpersonal stress, work and home overloads, network stressors) was associated with both negative and positive emotions on the same-day (i.e., reactivity) and next-day (i.e., residue). Over eight consecutive days, participants reported their daily negative and positive emotions, as well as exposure to and control over stressful experiences. After adjusting for age and gender, two-level multilevel models revealed a protective patterning of stressor control for dampened affective reactions to daily stressors driven by stressor control domains specific to interpersonal interactions (ps<.05). Days when people perceived more control over their arguments or avoided arguments were related to less negative and positive affective reactivity (smaller increases/decreases in negative/positive affect, respectively) compared to days with less perceived control. Perceived control over daily work stressors and home stressors were associated with dampened negative and positive affective reactivity, respectively. No associations between perceived stressor control and affective residue were significant. Results underscore the import of perceived stressor control for interpersonal stressors and the utility of perceived control as a protective factor for the effects of arguments and avoided arguments. Individuals may aim to leverage additional resources to feel more in control of their own daily arguments and avoided arguments.

